# Demographics, Clinical Characteristics, and Management of Idiopathic Intracranial Hypertension in Kuwait: A Single-Center Experience

**DOI:** 10.3389/fneur.2020.00672

**Published:** 2020-08-11

**Authors:** Jasem Youssef Al-Hashel, Ismail Ibrahim Ismail, Mohamed Ibrahim, John K. John, Fatemah Husain, Walaa Ahmed Kamel, Raed Behbehani, Samar Farouk Ahmed

**Affiliations:** ^1^Department of Neurology, Ibn Sina Hospital, Kuwait City, Kuwait; ^2^Department of Medicine, Faculty of Medicine, Health Sciences Centre, Kuwait University, Kuwait City, Kuwait; ^3^Department of Ophthalmology, Al-Bahar Eye Center, Kuwait City, Kuwait; ^4^Department of Neurology, Beni-Suef University, Beni Suef, Egypt; ^5^Department of Neurology and Psychiatry, Minia University, Minya, Egypt

**Keywords:** idiopathic intracranial hypertension, epidemiology, clinical characters, management, Kuwait

## Abstract

**Background:** Idiopathic intracranial hypertension (IIH) affects predominantly obese females during their reproductive age period. The demographics of this condition has not been studied in Kuwait before.

**Objectives:** To determine the demographics, clinical features, risk factors, and treatment modalities of IIH in the main neurology tertiary referral hospital in Kuwait and to compare our data with literature.

**Methods:** A retrospective study was conducted to identify cases of IIH seen between January 1, 2018, and December 31, 2018. Patients were diagnosed in and referred from the neurology and neuro-ophthalmology clinics.

**Results:** Our cohort consisted of 139 patients. We estimated a crude annual incidence rate of IIH of 3.28 per 100,000 population. Female-to-male ratio was 9.6:1. Mean age was 32.1 ± 10.8 years. Mean age of males was 31.46 ± 12.63 and that of females was 32.11 ± 10.67. The median of the duration from the first symptoms till diagnosis was 6 weeks (2–10 weeks). Headache was the most common symptom in 134 (96.4%) patients, followed by visual disturbances in 85 (61.2%) patients, transient visual obscurations (TVOs) in 84 (60.4%) patients, pulsatile tinnitus in 72 (51.8%) patients, diplopia in 22 (15.8%) patients, other symptoms (e.g., nausea, vomiting, radicular neck, and back pain) in 19 (13.7%) patients, and 1 (0.7%) patient had facial weakness. High body mass index (BMI) was seen in 89.9% of patients, either overweight or obese, and it was the most common risk factors in both males (46.2%) and females (61.1%). Anemia was found in 38.1%; 21.6% of patients used OCPs and 7.9% used vitamin A. Bilateral transverse sinus stenosis (BTSS) was detected in 47 (33.8%) patients. Only 2 (1.4%) patients developed “fulminant IIH” characterized by rapidly progressive disease. All the patients received medical treatment and only 12 (8.6%) needed surgical management.

**Conclusion:** Incidence of IIH in Kuwait is similar to other regional studies but higher than Western studies. Demographics and clinical features of IIH in our study are comparable to international and regional figures. Most of our patients had a benign course. IIH is more prevalent in females and strongly associated with obesity.

## Introduction

Idiopathic intracranial hypertension (IIH) is a condition characterized by increased intracranial pressure (ICP) with no evidence of space-occupying lesion, meningeal inflammation, or venous thrombosis. It was originally, described by Dandy as “pseudo-tumor cerebri” because of common clinical signs of intracranial hypertension in the absence of tumoral causes (1).

IIH has been strongly associated with obesity and female gender in reproductive age. Symptoms include headache, transient visual obscuration, pulsatile tinnitus, diplopia, and back and neck pain. Papilledema can lead to permanent visual loss if the condition is not treated appropriately in a timely manner. The diagnosis can be delayed since the clinical presentation and symptoms are variable and non-specific (2). The modified Dandy criteria used for diagnosis of IIH include the presence of raised ICP, typically papilledema, normal magnetic resonance imaging (MRI), and normal cerebrospinal fluid (CSF) composition with elevated opening pressure. In the absence of papilledema, the diagnosis is challenging and requires specific neuroimaging criteria to be met. Diagnostic criteria were recently revised by Friedman et al. (3). Fulminant IIH is defined as the acute onset of symptoms and signs of intracranial hypertension (<4 weeks between onset of initial symptoms and severe visual loss) with rapid worsening of visual loss over a few days (4).

The Idiopathic Intracranial Hypertension Treatment Trial (IIHTT) showed a beneficial effect of acetazolamide and weight loss in patients with mild visual loss (5).

Due to the paucity of large epidemiological, community-based studies, the incidence, prevalence, and clinical characterization of IIH are variable in literature. We studied the demographics, clinical characteristics, risk factors, and management modalities in a hospital-based population of IIH cases in Kuwait.

## Methods

We conducted a retrospective study of patients diagnosed with IIH in Ibn Sina Hospital, the only tertiary neurology, and neuro-ophthalmology referral hospital in Kuwait from January 1, 2018, to December 31, 2018.

The state of Kuwait has a population of 4.226 million (2018) with six governmental divisions and six major general hospitals. The incidence was defined as the number of new cases diagnosed with IIH (incidence of diagnosis) per 100,000 individuals.

All the medical records in Ibn Sina Hospital at the time of the study were reviewed. Lists of all patients discharged from the hospital with a primary diagnosis of IIH were recorded. Only patients who were diagnosed as IIH according to the revised diagnostic criteria were included (3).

All patients with suspected IIH undergo magnetic resonance imaging and venography (MRI/MRV) to rule out secondary causes of elevated intracranial pressure. All patients had sagittal and axial T1-weighted, T2-weighted and FLAIR MRI of the brain and either phase contrast or time of flight MRV using a three-dimensional phase contrast technique with 40 cm/s velocity encoding (time of repetition 25 ms; flip angle 20°; time of echo 7.2 ms) on either 1.5-T or 3-T machine.

Lumbar puncture (LP) and CSF manometry were also performed for all suspected IIH cases. LP was performed in the left lateral recumbent position and CSF opening pressure was measured with the legs extended. LP opening pressure is considered to be high if it is equal to or above 250 mm H_2_O. CSF was analyzed for biochemical and cytological composition. Neuro-ophthalmological evaluation included Snellen visual acuity, refraction, color vision, visual field tests with the Humphrey Field analyzer (HFA), and optical coherence tomography (OCT). Weight and height were measured and recorded in order to calculate body mass index (BMI, weight [kilograms]/height [square meters]).

Data on age at onset of symptoms, gender, height, weight, BMI, presence of comorbid conditions, medication use, findings of ophthalmic examination, neurological assessment, CSF opening pressure, radiological signs on cerebral MRI (prominent subarachnoid space around the optic nerves, flattening of the posterior sclera, partial empty sella, and slit-like ventricles), presence of BTSS on MRV, and type of medical or surgical treatment were recorded from the medical files.

The study was approved by the Institutional Review Board Committee of Ministry of Health of the State of Kuwait.

## Statistical Analysis

We used SPSS software for Windows, Version 23 (SPSS Inc., Chicago, IL, USA) to calculate range, mean, standard deviation, frequency, and percentage for different variables. The crude incidence rate per 100,000 persons was calculated using the total number of IIH cases included as numerator and the total number of Kuwaiti population as denominator. Ninety-five percent confidence intervals (CIs) were computed. Population data, based on the census carried out in 2018, were obtained from Kuwait Bureau of Statistics to determine the number of people at risk.

Chi-square test was adopted for categorical variables. Results were expressed by mean ± standard deviation, median with interquartile range (IQR), or percentage.

## Results

**Figure 1:**
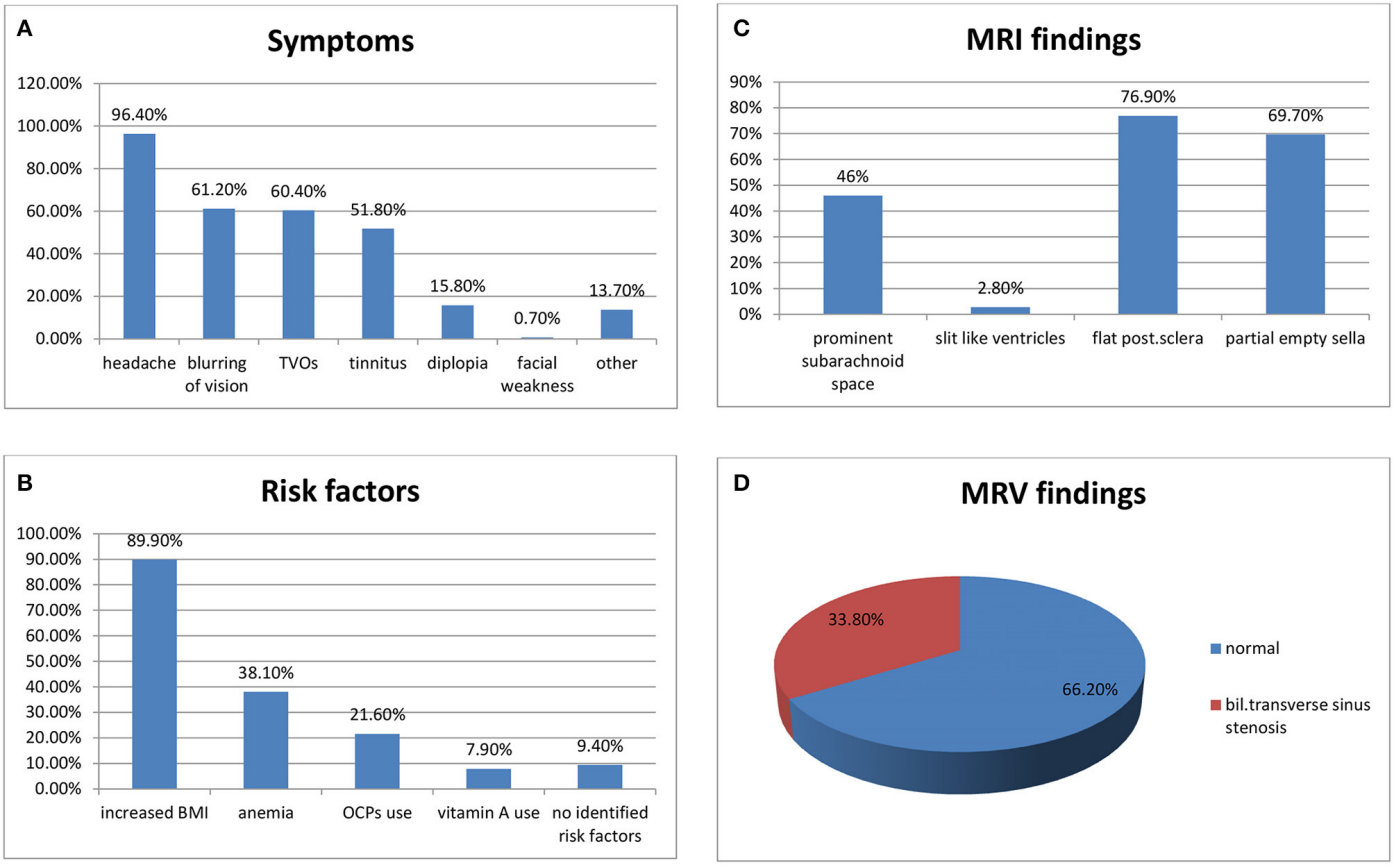
**(A)** Symptoms of IIH in our cohort, **(B)** risk factors, **(C)** MRV findings, and **(D)** MRI findings.

A total of 139 patients were included in our study during the assigned period. We estimated a crude annual incidence rate of IIH of 3.28 per 100,000 population (95% CI, 2.79–3.87). Demographics and clinical characteristics of our cohort are demonstrated in Table 1 and Figure 1.

**Table 1:** Demographic and clinical characteristics of IIH patients (*n* = 139).

**Variables**	**Mean (range), *N* (%)**
**Gender**	
Male	13 (9.4)
Female	126 (90.6)
**Age (years)**	32.1 ± 10.8 (12–59)
**Family history**	4 (2.8)
**Weight**	85.5 ± 17.2
**Height**	162.4 ± 6.9
**BMI**	
<18.5 (underweight)	0 (0.0)
18.5 to <25 (normal)	14 (10.1)
25 to <30 (overweight)	40 (28.8)
>30 (obese)	85 (61.1)
**Nationality**	
Kuwaiti	89 (64.0)
Egyptian	21 (15.1)
Indian	10 (7.2)
Others	29 (20.8)
**Symptoms at presentation**	
Headache	134 (96.4)
TVOs	84 (60.4)
Tinnitus	72(51.8)
Diplopia	22 (15.8)
Blurring of vision	85 (61.2)
Other symptoms	19 (13.7)
**Signs at presentation**	
Papilledema (*n* = 139)	138 (99.3)
Sixth N. (*n* = 139)	22 (15.8)
Seventh N. (*n* = 139)	1(0.7)
**Risk factors**	
BMI > 25	125 (89.9)
No identifiable risk factors	13 (9.3)
**Comorbidities**	
Anemia (*n* = 139)	53 (38.1)
PCOS (*n* = 139)	13 (9.4)
OCP (*n* = 139)	30 (21.6)
Vitamin A (139)	11 (7.9)
Hypertension	27 (19.4)
Diabetes mellitus	11(7.9)
Migraine	7 (5.0)
Hypothyroidism	6 (4.3)
**Treatment**	
Medical treatment	139 (100)
CSF shunt	5 (3.6)
ONSF	7 (5.0)
VSS	1 (0.7)
Bariatric surgery	10 (7.1)

*Qualitative data were described using number and percent. Normally quantitative data were expressed as mean ± SD. BMI, body mass index; TVOs, transient visual obscuration; PCOS, polycystic ovary syndrome; OCP, oral contraceptive pills; CSF, cerebrospinal fluid; ONSF, optic nerve sheath fenestration; VSS, venous sinus stenting*.

### Demographics

One hundred twenty-six (90.6%) were females and 13 (9.4%) were males with a female-to-male ratio of 9.6:1. Family history was positive in 4 patients (2.8%).

Mean age of the patients was 32.1 ± 10.8 years, ranging from 12 to 59 years.

Mean age of males was 31.46 ± 12.63 and that of females was 32.11 ± 10.67 years.

The duration from the first symptoms till diagnosis ranged from 2 to 10 weeks with a median of 6 weeks.

Most of the patients were of Kuwaiti nationality [89 (64.0%)], followed by Egyptian nationality [21 (15.1%)] and Indian nationality [10 (7.2%)], and 29 (20.8%) were from other nationalities.

### Clinical Characteristics

Headache was the most common presenting symptom in 134 (96.4%) patients. On a scale of 0–10, the mean of headache severity was 6.5. It was described as classical high-pressure headache in 49 (35.2%) patients, dull aching, holocranial, or pressure-like in 37 (26.6%), chronic daily headache in 31 (22.3%), and migraine-like in 22 (15.8%) patients.

Blurring of vision was present in 85 (61.2%) patients and 2 (1.4%) patients developed “fulminant IIH” and visual loss. Transient visual obscuration (TVOs) were reported by 84 (60.4%) patients, pulsatile tinnitus was reported by 72 (51.8%) patients, diplopia was reported by 22 (15.8%) patients, and 19 (13.7%) patients reported other symptoms (e.g., nausea, vomiting, radicular neck, and back pain). One (0.7%) patient had left facial weakness (Figure 1).

Neurological examination upon presentation showed bilateral papilledema in 138 (99.3%) patients and only 1 patient with normal fundus exam. Papilledema was mild in 48 (34.8%) patients, moderate in 60 (43.5%) patients, and severe in 30 (21.7%) patients. Seventy-eight (56.1%) patients had a visual acuity of 20/20, 7 (5.0%) patients with 20/400, 2 (1.4%) patients with 20/800, 2 (1.4%) patients with “fulminant IIH” with no perception of light, and 50 (35.9%) patients in between. Sixth nerve palsy was found in 22 (15.8%) patients, of whom 4 (2.8%) had bilateral palsy. One patient (0.7%) had left lower motor neuron facial nerve palsy.

Visual acuity, mean deviation of the visual fields (MD), and the retinal nerve fiber layer thickness (RNFLT) obtained by optical coherence tomography (OCT) at presentation are shown in Table 2. The mean VA logMAR was 0.1 (0.1–3) in the right eye and 0.1 (0–3) in the left eye. The mean RNFLT was 125 μm (61–403) in the right eye and 137 μm (87–405) in the left eye. Visual field mean deviation (MD) was evaluated by HFA and was −3.8 (−30.1 to −0.4) for the right eye and −4.3 (−33.9 to −0.3) for the left eye (Table 2).

**Table 2:** Visual assessment analysis of the IIH patients (*n* = 139).

	**Right**	**Left**
VA (logMAR)	0.1 (0–1.3)	0.1 (0–3)
VF MD	−3.8 (−30.1 to −0.4)	−4.3 (−33.9 to −0.3)
RNFLT	125 (61–403)	137 (87–6477)

*Abnormally distributed data were expressed using Median (Min.–Max.)*.

*VA: Snellen visual acuity converted to LogMAR score (LogMAR 0 = 20/20 and LogMAR 1 = 20/200)*.

*VFMD, visual field mean deviation (decibels)*.

*RNFLT: Retinal nerve fiber layer thickness obtained by spectral-domain optical coherence tomography (microns)*.

### Risk Factors

BMI ranged from 19.4 to 58.6. None of the patients had BMI <18.5 (underweight). BMI of 14 patients were between 18.5 and <25 (normal), 40 (28.8%) were between 25 and <30 (overweight), and 85 (61.1%) patients had a BMI of more than 30 (obese). Thirteen (9.4%) patients had no identifiable risk factor despite full workup.

### Comorbidity

Anemia was found in 53 (38.1%) patients. Abdominal ultrasonography was done for all our female patients and polycystic ovary (PCO) was found in 13 (9.4%) patients. Thirty (21.6%) patients were taking oral contraceptive pills while 11 (7.9%) patients reported taking vitamin A (retinol) and isotretinoin for acne treatment.

Other common comorbidities among our cohort were hypertension in 27 (19.4%), diabetes mellitus in 11 (7.9%), migraine in 7 (5.0%), and hypothyroidism in 6 (4.3%) patients.

The CSF opening pressure ranged from 250 to 990 mmH_2_O with a mean of 360 mmH_2_O. Closing pressure ranged from 120 to 500 mmH_2_O with a mean of 200 mmH_2_O. CSF analysis was within normal range for all patients for proteins, 229 mg/dl (100–630); glucose, 3.4 mmol/L (2.3–4.5); and cells, <5 white blood cells. LP needed to be repeated in 28 (20.1%) patients through their course of illness.

Post-LP headache was reported in 32 (23.0%) patients during the first week after CSF tapping.

MRI of the brain was done for all our patients; prominent subarachnoid space around the optic nerves was found in 64 (46.0%) patients, flattening of the posterior sclera in 107 (76.9%), partial empty sella in 79 (69.7%), and slit-like ventricles were seen in only 4 (2.8%) patients (Figure 1).

MRV was done for all our patients. Cases with cerebral venous thrombosis were excluded from this cohort. MRV was normal in 92 (66.2%) patients, and it showed bilateral transverse sinus stenosis (BTSS) in 47 (33.8%) patients (Figure 1).

### Treatment

Most of our patients, 127 (91.4%), were managed successfully with weight loss and medical therapy, while only 12 (8.6%) patients needed surgical management.

Acetazolamide in a dose ranging from 1 to 4 g/day was given to 123 (88.4%) and was well-tolerated. Topiramate was given to 31 (22.3%) patients. Furosemide was given to 5 (3.6%) patients, chlorthalidone to 1 (0.7%) patient, and corticosteroids to 1 (0.7%) patient. Four patients (2.8%) received combination therapy of acetazolamide and topiramate.

Seven (5.0%) patients underwent optic nerve sheath fenestration (ONSF), 5 (3.6%) patients underwent CSF shunting (4 ventriculoperitoneal and 1 lumboperitoneal shunt), and 1 (0.7%) patient underwent endovascular venous sinus stenting (VSS). Ten (7.1%) patients in our cohort underwent bariatric surgery in addition to medical treatment.

### Female Predisposition

There was no significant difference in the clinical and demographic characteristics between male and females. High BMI was the most prevalent risk factor in both genders. Intake of vitamin A was reported only in the female cohort while anemia was reported in both cohorts.

## Discussion

IIH is primarily a disorder of young obese women with a worldwide incidence around 12–20 per 100,000 people per year in obese females at childbearing period, but only 0.5–2 per 100,000 people per year in the general population (6). In this study, by studying the referrals to the main tertiary neurology hospital in Kuwait, we have estimated a crude annual incidence rate of IIH of 3.28 per 100,000 population.

Few studies have addressed the epidemiology of IIH in the Arab countries and Middle East. One retrospective study from Oman (7) evaluated 40 patients during a period of 11 years, another study from Saudi Arabia (8) included 99 patients, a study from Dubai (UAE) (9) reported 50 patients, a study from Libya (6) reported 81 patients in a 7-year period, and one from Israel reported 428 patients from 2005 to 2007 (10). The incidence of IIH in Middle East countries was found to be higher than the Western rates and has been estimated at 2.02–2.2/100,000 in the general population compared to 1.6 per 100,000 in the Western general population (11). The variation in incidence from country to country can be attributed to the variation in obesity prevalence among different populations. There has been an increase in obesity prevalence in both genders that contributes to the increasing incidence of IIH in these countries (12).

Females constituted the majority of the population in our cohort (90.6%), with a female-to-male ratio of 9.6:1, which is similar to other published studies that found a ratio from 4:1 to 8:1 (13, 14). A large series by Keslar et al. confirmed that only about 10% of IIH patients are men. Unlike women, men tended to be older at presentation and obesity was not found to be a significant risk factor as in women, which we did not find in our study (15).

The mean age in our study was 32.1 ± 10.8 years (range, 12–59), which is similar to Thambisetty et al. (4), reporting in the IIHTT with a mean age of 29.0 ± 7.4 years (range, 18–52 years).

Family history was positive in 4 patients (2.8%) in our cohort who were all obese. The positive family history percentage varies in the literature between 5.0% in IIHTT participants and 11.3% in the study of Corbett (16). IIH could have a true genetic predisposition, but some of those patients could have genetic predisposition to obesity as well. In a study about the prevalence of papilledema in asymptomatic obese patients, Krispel et al. (17) found papilledema in only 0.3% of patients.

Symptoms in our cohort were similar to those found in other previous studies (4, 18). Headache was the most common initial symptom in our patients (96.4%) (18, 19). Friedman et al. (20) found that the commonest phenotype was migraine (52%) followed by tension-type headache (22%) unlike our study where migraine constituted only 15.8% and the commonest phenotype in our cohort was the classical high-pressure headache in 35.2% of patients. In about half of IIHTT participants, headache was constant or daily (4) while only 22.3% of our patients had chronic daily headache.

TVOs are transient episodes of monocular or binocular visual loss usually during postural changes and lasting <30 s followed by full visual recovery. They were reported by 60.4% of our patients compared to 68% in IIHTT participants or 72% in another study (4, 21).

Tinnitus in IIH is usually bilateral and pulsatile in nature. It was reported by 51.8% of our patients similar to several studies that found tinnitus in 50–60% of their cases (2, 22).

In our cohort, 15.8% developed diplopia secondary to either unilateral or bilateral sixth cranial nerve palsy as a false localizing sign (23). In literature, diplopia was reported in 18% of IIHTT participants while it was in 30% in other studies (24, 25).

Seventh nerve palsy was seen in one female in our cohort, and it has been reported in the context of IIH in literature in 2–6% of cases (26, 27). The pathophysiology of facial palsy in IIH is not known but likely represents a pressure-related phenomenon. The seventh nerve run only a short course in the subarachnoid space before entering the petrous temporal bone and is relatively protected from the effects of elevated pressure (28). Two reports (28, 29) related it to elevated ICP in the posterior fossa and enlargement of fallopian canals and it usually resolves after lowering ICP.

Papilledema was found in almost all our patients and one patient only had IIH without papilledema (IIHWOP) who was diagnosed based on elevated opening pressure and neuroimaging criteria. Digre et al. (30) reported IIHWOP in 5.7% of their 353 IIH patients. Papilledema was bilateral in all our cases; however, it can be unilateral in 3.6–10.0% of IIH patients (31).

Obesity has a strong association with IIH in most cases and weight loss alleviates IIH signs and symptoms significantly while weight gain is linked to recurrence (32). Kuwait ranks first among Middle East countries in obesity prevalence (55.2%) followed by Egypt (48%) and United Arab Emirates (UAE) (42%). These percentages are higher than European countries and comparable to the US (48.3%) (11). It was the commonest risk factor in our study (90%), which is comparable to Western published data (4), while data from other Middle East countries estimated the prevalence between 60 and 80% (11).

We reported in our cohort anemia, use of OCPs, vitamin A, and PCOS, which are common among women of childbearing age. In our community, consumption of vitamin A by young ladies is common and may have an impact on prevalence of IIH in our cohort. Vitamin A intake was reported in previous studies of IIH prevalence and morphology (11). There is a clear association between vitamin A intoxication and intracranial hypertension. It is hypothesized that the pathogenesis of IIH involves abnormal vitamin A metabolism. However, if altered vitamin A metabolism is associated with IIH, there must be other factors influencing the disease (33). However, some authors prefer the term “pseudotumor cerebri syndrome” in this situation (33).

Hypertension and DM were other common comorbidities in more than a quarter of our cohort. These diseases have high prevalence in obese patients, and this was similar to a recent study from Australia where hypertension was found in 22.7% of IIH patients. However, the highest comorbidities they found were migraine (31.8%) and depression (30%) (34). Prevalence of hypertension and DM in Kuwait ranged from 25 to 35% (35).

BTSS was found in 33.8% of our cohort. This finding is much lower than other studies that found BTSS prevalence in the 90% range (36–38). This can be related to the technique used to demonstrate BTSS, as MRV, 2D-TOF images, and 3D-PC images acquired with a VENC of 40 cm/s tend to bias the interpretation of flow signal toward normality, underestimating the disturbances of flow of transverse sinuses, while 3D-PC MRV set with the VENC to 15 cm/s represents the best non-invasive technical approach for visualizing BTSS in patients with IIH (39).

Weather venous stenosis playing a role in the pathophysiology of elevated pressure is still controversial. However, venous sinus stenting has been shown to be beneficial in IIH in some case series, but its long-term benefits are still unknown (40). We had only one patient who underwent VSS with a good outcome but this cannot be generalized.

Most of our patients (91.4%) needed only medical treatment, which is in line with a recent UK study that reported 91.6% of responders to medical treatment (41).

## Strength of the Study

Our study is the first one that estimated the incidence of IIH in Kuwait. There is only one tertiary hospital in Kuwait that takes care of patients requiring neurology and neuro-ophthalmology care. We collected the required information to calculate the incidence of IIH in Kuwait. This study represents the first comprehensive description of a large IIH cohort from Kuwait.

## Limitations of the Study

First, being a retrospective study conducted in a single hospital-based population, the results are subjected to bias and lack of generalization. Second, the underreporting of BTSS in our cohort can be attributed to the technique used in MRV rather than actual low prevalence. Another limitation of the study is including cases who have been taking vitamin A, which, according to some authors, should not be considered “idiopathic” and the term “pseudotumor cerebri syndrome” would be more plausible in this situation.

## Summary of Main Findings of the Results

Crude annual incidence rate of IIH is 1.6 per 100,000 population. Female-to-male ratio was 9.6:1. Mean age was 32.1 ± 10.8 years. Headache was the most common symptom in 134 (96.4%) patients. High BMI was seen in 89.9% of patients, either overweight or obese, and it was the most common risk factor in both males (46.2%) and females (61.1%). BTSS was detected in 47 (33.8%) patients. All the patients received medical treatment and only 12 (8.6%) needed surgical management.

## Conclusion

Incidence of IIH in Kuwait is high in comparison to Western countries and this is in line with increasing obesity rates in our region. The clinical presentation, risk factors and management of IIH in our study are comparable to the international and regional studies of IIH. Most of our patients had benign course. IIH is more prevalent in females and strongly associated with obesity.

## Data Availability Statement

All datasets generated for this study are included in the article/supplementary material.

## Ethics Statement

This study was approved by the Institutional Review Board Committee of Ministry of Health of the State of Kuwait.

## Author Contributions

All authors collected and analyzed the data and wrote and reviewed the manuscript.

## Conflict of Interest

The authors declare that the research was conducted in the absence of any commercial or financial relationships that could be construed as a potential conflict of interest.
